# In Vitro Comparison of Microenvironmental pH Modulation Approaches for Minzasolmin

**DOI:** 10.3390/pharmaceutics18101243

**Published:** 2026-09-30

**Authors:** Fanny Stauffer, Anne Bouquelle, Sarah Le Meur, Domagoj Segregur, Faiza Laredj, Célal Ates, Christophe Huygens, Gabrielle Pilcer

**Affiliations:** 1 Pharmaceutical Development Sciences, UCB, Chemin du Foriest 1, 1420 Braine l’Alleud, Belgium; sarah.lemeur@ucb.com (S.L.M.); domagoj.segregur@ucb.com (D.S.); christophe.huygens@ucb.com (C.H.); gabrielle.pilcer@ucb.com (G.P.); 2 Analytical Development Sciences, UCB, Chemin du Foriest 1, 1420 Braine l’Alleud, Belgium; anne.bouquelle@ucb.com; 3 Patient Supply Portfolio Management, UCB, Chemin du Foriest 1, 1420 Braine l’Alleud, Belgium; faiza.laredj@ucb.com; 4 Drug Substance Process Sciences, UCB, Chemin du Foriest 1, 1420 Braine l’Alleud, Belgium; celal.ates@ucb.com

**Keywords:** formulation, solubility enhancement, microenvironmental pH, dissolution, bioavailability, patient-centricity

## Abstract

**Background/Objectives:** Minzasolmin is a weakly basic drug developed as a potential therapy in Parkinson’s. Amongst the strategies that have been developed to enhance API solubility, pH modification is an effective and patient-centric approach that improves solubility by creating a microenvironmental pH around the drug. The objective of this study was to evaluate different formulation approaches to develop an immediate-release tablet or capsule containing fumaric acid as a pH modifier. **Methods:** Ten formulations were developed, varying the degree of contact between the API and pH modifier. These formulations were then assessed in three biorelevant dissolution conditions to estimate their robustness towards variations in gastric pH. **Results:** In highly buffered conditions, only the over-encapsulated tablet showed a clear improvement in the dissolution profile, with up to 5.1 times the percentage dissolved compared to the capsule without acid and up to 1.4 times compared to the target granule-in-capsule with acid. In low-buffered conditions, though, all prototypes with acid showed an improvement in the percentage dissolved, between 54 and 79 times compared to that of the capsule without acid, regardless of the degree of contact between the organic acid and the API. This was associated with a decrease in pH to 5 in the gastric compartment. **Conclusions:** These results suggested that the acidic microenvironment, estimated via the bulk pH, and hence the dissolution rate may not require such a high degree of contact between the API and the pH modifier. Based on in vitro results, the over-encapsulated tablet as well as prototypes with a lower degree of contact between the acid and PI will be evaluated in vivo in a future study to evaluate the bio-relevance of the approach.

## 1. Introduction

Minzasolmin is a small-molecule inhibitor of α-synuclein misfolding, developed as a potential disease-modifying therapy in Parkinson’s [1]. In the BCS (Biopharmaceutical Classification System) [2], minzasolmin is classified as a BCS class II active pharmaceutical ingredient (API) due to its low solubility. It is indeed a weak basic compound with pH-dependent solubility (i.e., thermodynamic solubility ≥100 mg/mL at pH 1.6 and about 0.01 mg/mL at pH 7.0). As such, minzasolmin bioavailability is expected to be influenced by variations in gastric pH.

It has been estimated that almost 27% of Parkinson’s patients suffer from acid reflux [3]. As a consequence, about 25% of patients are treated with acid-reducing agents [4], thereby increasing gastric pH. Gastric pH variability is also well described in the literature as being influenced by several physiological factors, such as food effect [5,6,7], specific patient populations due to reduced acid secretion [8], disease [9,10], ethnicity [11] or age [12]. These populations may experience elevated gastric pH, also referred to as hypo- or achlorhydria (i.e., too low or no gastric acid secretion). Considering the high number of factors leading to gastric pH variability, it was therefore decided to evaluate formulation approaches to ensure suitable solubilization of the API in the gastrointestinal tract.

Strategies to mitigate the effect of high gastric pH on bio-performance include co-administration with acidic beverages, pre-treatment with organic acid, development of solid dosage formulations containing organic acid and the development of enabling formulations [13]. The first two strategies are considered less patient-centric as they require an additional action from the patient and were discarded [14,15]. Enabling formulation approaches include chemical approaches (e.g., co-solvents, solubilising agents), crystal engineering (e.g., salt or cocrystal formation) or physical approaches (e.g., amorphous solid dispersion, cyclodextrin complexation, lipid-based formulation, particle size reduction) [16]. Such development can greatly increase the development timelines. pH modification is an effective and patient-centric strategy to improve solubility by creating a microenvironmental pH around the drug [17,18]. The microenvironmental pH can be described as the pH of the saturated solution in the immediate vicinity surrounding the drug particle [19]. With decreasing microenvironmental pH, drug solubility increases and drug release is enhanced.

A few studies reported the use of organic acids added to a formulation to overcome elevated gastric pH. Onoue et al. described the use of fumaric acid inside a granule formulation to improve the bioavailability of dipyridamole under hypochlorhydria [20]. Badawy et al. evaluated 11 formulations containing tartaric acid (intra- and extra-granular), citric acid, succinic acid or cyclodextrins [21]. Based on their study, the bioavailability of BMS-56389 could be improved by using tartaric acid in the extra-granular phase. Pu et al. also performed the screening of organic acids and surfactants to reduce the pharmacokinetic variability of a weakly basic drug compound, showing reduced variability with citric acid and sodium lauryl sulfate [22]. Similarly, Mitra et al. compared the bioavailability of capsule formulations with and without citric acid added either in the extra-granular phase or co-melted with compound A [23]. In their study, the highest performance was obtained by citric acid co-melted with the API. The authors attributed this result to the intimate contact between the API and pH modifier. Taniguchi et al. evaluated 10 organic acids in granule formulations containing dipyridamole considering manufacturability, product stability and bio-performance [24]. This study concluded that p-toluene sulfonic acid monohydrate met the three criteria. This study nonetheless highlighted that organic acids and the weak base are likely to interact, limiting product stability. These studies exemplify the difficulty of developing a manufacturable, stable, and yet performant formulation using organic acids. Taniguchi et al. especially highlighted that the level of microenvironmental pH and its duration in vivo can be key factors for improving drug dissolution [25]. Advanced formulation strategies were therefore developed to prevent the pH modifier from leaching out of the formulation, such as film-coated pellets containing organic acids [26,27]. To conclude, the way to incorporate a pH modifier in a formulation is expected to affect the drug product performance.

This study reports the formulation development of an immediate-release oral solid dosage form (tablet or capsule) to enhance the bioavailability of minzasolmin. A granule-in-capsule (GiC) formulation containing fumaric acid in the extra-granular phase was evaluated in a previous pharmacokinetic study in pre-treated dogs as proof of concept. A 2.4-fold increase in the Cmax was achieved compared to the GiC formulation without fumaric acid [28]. Based on this proof of concept, biorelevant dissolution tests were developed, and different formulation strategies for incorporating a pH modifier in the formulation were evaluated. The goal was to maximise the dissolution of the drug in biorelevant media with acceptable amounts of pH modifier to ensure manufacturability, product stability, and reduced capsule/tablet size for improved patient experience.

## 2. Materials and Methods

### 2.1. Materials

Minzasolmin was characterised as a weakly basic BCS class II molecule with a pH-dependent solubility profile. Lactose monohydrate (Pharmatose 200, DFE pharma, Nijmegen, The Netherlands) and microcrystalline cellulose (MCC, Avicel PH102, Dupont, Cork, Ireland) were used as fillers. Sodium croscarmellose (Ac-Di-Sol, Dupont, Cork, Ireland) was used as a disintegrant. Colloidal silicon dioxide (Aerosil 200, Evonik, Rheinfelden, Germany) was used as glidant. Hydroxy-propyl cellulose (HPC, Klucel EF, Ashland, Hopewell, VA, USA) was used as a binder. Magnesium stearate (Ligamed MF 2V, Peter Greven, Venlo, The Netherlands) was used as a lubricant.

Pre-tests were performed to select the most suitable organic acid. Eight organic acids were screened using two-stage biorelevant dissolution tests under achlorhydric conditions to evaluate the ability of the acid to overcome pH-dependent dissolution of minzasolmin. These organic acids were also evaluated for their propensity to induce solid-state change of the API (e.g., polymorphic change, salt formation) at various molar ratios. Finally, compatibility tests were performed to evaluate the risk of chemical degradation in the binary mixture. Based on these tests, fumaric acid (Merck, Germany) was selected as a pH modifier as it showed good manufacturability, the best stability and maximised the bio-performance in pre-treated dogs [28]. Hypromellose capsules (VCaps, Capsugel, France) of diverse sizes were used for encapsulated prototypes.

### 2.2. Prototype Design

Ten prototypes were developed to evaluate the impact of the degree of contact between fumaric acid and the API on in vitro dissolution performance. Indeed, a high degree of contact is expected to improve bio-performance, but also to negatively affect the stability of the drug product. All prototypes were individually optimised to ensure manufacturability while limiting formulation variations that could interfere with comparability (see Section 2.2.10). Table 1 provides a brief description of the developed formulations.

#### 2.2.1. Granule Manufacturing

The drug product process was based on wet granulation, followed by final blending and either capsule filling or tabletting. Based on pre-tests, it was decided to add the fumaric acid in the extra-granular phase. The granule composition was therefore the same for all tested formulations and was composed of 40.00% API, 44.92% lactose monohydrate, 6.36% MCC, 2.22% sodium croscarmellose, 0.50% colloidal silicon dioxide and 6.00% HPC. The same batch of granules was used in all formulations.

Raw materials were first manually sieved on a 2 mm screen and poured into a 30 L bin (Servolift GmbH, Offenburg, Germany). Materials were then blended for 15 min at 15 rpm. The obtained pre-blend was then granulated using twin-screw granulation (Consigma-1, GEA, Wommelgem, Belgium). Granulation parameters were optimised to ensure that suitable granule attributes were achieved. The granulator was mounted with a 2 × 6-SCE screw (i.e., two kneading zones of six kneading elements with a stagger angle of 60° and an additional zone with size control elements to reduce fines). The feeder was set to 16 kg/h, the screw speed to 650 rpm, the liquid-to-solid ratio to 40% and the barrel temperature to 25 °C. Wet granules were then dried using a GPCG2 fluid-bed dryer (Glatt, Binzen, Germany). The inlet air temperature was set to 70 °C. The air flow rate was initially set to 100 m^3^/h and adapted based on fluidisation during drying. Drying was stopped when the residual moisture content of the granules fell below 1.5%. Dried granules were finally milled using an SLS U5 conical mill (Quadro, Waterloo, ON, Canada) mounted with a square 0.813 mm screen and mill speed set at 1750 rpm.

#### 2.2.2. Granule-in-Capsule Manufacturing (Prototypes 1 and 2)

Prototypes 1 and 2 were granule-in-capsule prototypes without and with fumaric acid, respectively. Both prototypes were used as references regarding in vitro dissolution performance, as they had previously been tested during a pharmacokinetic study in pre-treated dogs [28]. Prototype 1 (i.e., GiC without acid) was used as a baseline for biorelevant dissolution tests, as it was known to under-expose subjects with achlorhydria. Prototype 2 (i.e., GiC with acid) was used as a target for biorelevant dissolution tests, as it was known to improve exposure of subjects with achlorhydria.

Prototypes contained 225.0 mg of granules and 1.1 mg of magnesium stearate. Prototype 2 also contained 20 mg of fumaric acid. For both prototypes, excipient(s) were first manually sieved on a 1 mm screen. Granules and sieved excipient(s) were then poured into a T2G Turbula blender (WAB group, Nidderau, Germany). Blending was performed for 3 min at 22 rpm. Blends were manually filled into size 1 capsules. It has to be noted that only half of the target dose (i.e., 90 mg) could be fitted in a single size 1 capsule. These prototypes would therefore require two capsules to be administered to reach the target dose and/or larger capsule sizes, which may not be well accepted by patients.

#### 2.2.3. Capsule-in-Capsule Manufacturing (Prototype 3)

Prototype 3 was a capsule-in-capsule (CiC) formulation. It was assessed to physically separate the fumaric acid from the API, while still considering a capsule formulation. For this prototype, 20 mg of fumaric acid was inserted into a size 4 capsule. This capsule was inserted into a size 00 capsule, also containing the active blend. The active blend was composed of 225.0 mg of granules, 25.3 mg of MCC and 2.5 mg of magnesium stearate. The manufacturing process was similar to prototypes 1 and 2 (i.e., Turbula blending and manual encapsulation). As for prototypes 1 and 2, only half of the target dose (i.e., 90 mg) could be fitted in a single size 1 capsule; hence, the size 00 capsule was selected for testing, though it is not realistic in terms of patients’ acceptability.

#### 2.2.4. Tablet Manufacturing (Prototypes 4 and 5)

Prototypes 4 and 5 were monolayer tablet prototypes without and with fumaric acid, respectively. They were developed to compare with corresponding GiC prototypes (i.e., prototypes 1 and 2). Prototype 4 was composed of 450.0 mg of granules, 64.3 mg of MCC, 10.7 mg of sodium croscarmellose, 2.7 mg of silicon dioxide and 5.3 mg of magnesium stearate. The composition of prototype 5 was similar, as 40 mg of the MCC was replaced by 40 mg of fumaric acid.

For both prototypes, extra-granular phase excipients were manually sieved on a 1 mm screen. Granules and all excipients except magnesium stearate were blended using a T2G Turbula blender for 5 min at 22 rpm. Magnesium stearate was then added to the blend, and blending was extended for 3 min at 22 rpm. Tablets were produced using a Styl’one Evolution compaction simulator (Medelpharm, Beynost, France); 16 × 7.37 mm punches were used, allowing the intended dose of 180 mg of API in a single tablet of acceptable dimensions for swallowability. A pre-compression height of 6.5 mm and a compression height of 3.8 mm were applied, resulting in a tablet hardness of about 120 N and a tablet thickness of about 6.8 mm for both prototypes. It should be noted that tablets were also produced at 250 N to ensure that dissolution results in biorelevant media were not sensitive to tablet hardness, ensuring comparability between the different prototypes.

#### 2.2.5. Over-Encapsulated Tablet Manufacturing (Prototype 6)

Prototype 6 was an over-encapsulated tablet. For this prototype, it was hypothesised that dissolution or gastric liquid would enter the capsule shell before its disintegration and pre-dissolve the components of the tablet, creating a microenvironment saturated with acidifier inside the capsule shell.

For this prototype, the monolayer tablet with acid (prototype 5) was manually inserted into a size 0 capsule. Though the target dose could not fit into a single size 1 capsule, formulation optimisation and punch design were expected to be sufficient to achieve this goal. As this study aimed at screening different approaches to include the acidifier in the formulation, such optimisation was not performed.

#### 2.2.6. Bilayer Tablet Manufacturing (Prototype 7)

Prototype 7 was a bilayer tablet containing the API in one layer and the acid in the other layer. It was designed to physically separate the API from the acid while keeping a relatively close contact to enable the acidic microenvironment. This bilayer tablet was composed of an active layer containing 450.0 mg of granules blended with 122.5 mg of MCC, 2.4 mg of colloidal silicon dioxide and 5.1 mg of magnesium stearate; and an acidic layer composed of 40 mg of fumaric acid, 42.8 mg of MCC, 0.8 mg of sodium croscarmellose and 1.4 mg of magnesium stearate.

Excipients were manually sieved on a 1 mm screen. Both the active blend and the acidic blend were produced by blending all ingredients except the magnesium stearate for 5 min at 22 rpm using a T2G Turbula blender. Magnesium stearate was then added, and the blending was extended for 3 min at 22 rpm. Bi-layer tablets were produced using a Styl’one Evolution compaction simulator mounted with 14.48 × 8.13 mm punches. The compression started with the active layer, which was pre-compressed at 80 daN to achieve a layer hardness of 60 N. The acidic layer was fed on top of the active layer and compressed to reach a tablet hardness of 230 N, the hardness required to maintain tablet integrity during the friability test. As for previous tablet prototypes, the target dose of 180 mg of API could be achieved in a single tablet of acceptable dimensions for swallowability.

#### 2.2.7. Tablet-in-Tablet Manufacturing (Prototype 8)

Prototype 8 was a tablet-in-tablet (TiT) formulation containing the acid in the inner tablet (acid core) and the API in the external tablet (active shell). It was developed as an alternative to the bilayer tablet to keep the separation between the acid and the API while changing the kinetics of the microenvironment creation during dissolution.

The acid core was composed of 40 mg of fumaric acid, 41.0 mg of MCC, 0.8 mg of sodium croscarmellose and 0.4 mg of magnesium stearate. This acid core tablet was compressed into an active tablet shell composed of 450.0 mg of granules, 40.6 mg of MCC and 5.1 mg of magnesium stearate.

The active and acidic blends were manufactured similarly to prototype 7 (i.e., bilayer tablet). The TiT prototypes were manufactured using a Styl’one Evolution compaction simulator. Acid cores were first produced using various shapes. As no impact of the acid core shape was observed on dissolution rates, the 5.5 mm round punches targeting a hardness of 80 N were selected. To insert the acid core inside the active shell, a first layer of active blend was pre-compressed at 80 daN using 16 × 7.37 mm punches. One acid core was then centred on top of this first layer, and the matrix was filled with more active blend to reach the target weight. The powder was then compressed to reach a hardness of 165 N, the hardness required to maintain tablet integrity during the friability test. As for previous tablet prototypes, the target dose of 180 mg of API could be achieved in a single tablet of acceptable dimensions for swallowability.

#### 2.2.8. Mini-Tablets in Capsule Manufacturing (Prototype 9)

Prototype 9 was a mini-tablet-in-capsule formulation. It consisted of active mini-tablets containing the API and acid mini-tablets containing fumaric acid inserted into a capsule. This prototype was developed to obtain a physical barrier between the API and the acid, while enabling an acidic microenvironment inside the capsule shell.

The active mini-tablets were composed of 450.0 mg of granules, 78.0 mg of MCC and 4.7 mg of magnesium stearate. The acid mini-tablets were composed of 40.0 mg fumaric acid, 13.6 mg MCC, 0.8 mg sodium croscarmellose and 0.8 mg magnesium stearate. 532.7 mg of active mini-tablets and 55.2 mg of acid mini-tablets were encapsulated in a size 00 capsule.

The active and acidic blends were manufactured similarly to prototypes 7 and 8 (i.e., bilayer tablet and TiT). The mini-tablets were manufactured using a Styl’one Evolution compaction simulator mounted with 2.5 mm multi-tip punches targeting a 2.5 mm thickness. Acid and active mini-tablets were then manually filled into capsules. As for previous prototypes in capsule, the target dose could not fit into a single size 1 capsule; hence, the size 00 capsule was selected for testing, though it is not realistic in terms of patients’ acceptability.

#### 2.2.9. Tablet-in-Capsule Manufacturing (Prototype 10)

Prototype 10 was a tablet-in-capsule (TiC) formulation with fumaric acid as powder. It was developed to obtain a physical separation between the API and the acid, while trying to enable an acidic microenvironment inside the capsule shell. For this prototype, various acid/API molar ratios were evaluated during pre-tests, concluding that a higher acid/API ratio of 0.94 was needed to see an effect of the fumaric acid on dissolution. This was likely due to the reduced level of contact between the fumaric acid and the API. The monolayer tablet without acid (prototype 4) was manually inserted into a size 00 capsule with 169 mg of fumaric acid added as a powder. As for previous prototypes in capsule, the target dose could not fit into a single size 1 capsule; hence, the size 00 capsule was selected for testing, though it is not realistic in terms of patients’ acceptability.

#### 2.2.10. Prototype Comparison

As described above, prototypes varied in terms of dimensions (e.g., capsule size or tablet dimensions). Pre-tests were performed showing no impact of these parameters on the dissolution rates. Tablet prototypes were also evaluated at different tablet hardness/porosity, showing no impact of this attribute on dissolution rates within the range of selected prototypes (i.e., 100–250 N). For some capsule prototypes, it was not possible to fit the target dose into a single capsule. Dissolution pre-tests were also performed, demonstrating that the dissolution profile of two 90 mg units was equivalent to one 180 mg unit. The composition of the different prototypes was kept as identical as possible. However, some excipients were needed to achieve suitable manufacturability. Finally, all formulations containing fumaric acid, except for prototype 10, targeted an acid/API molar ratio of 0.22, which was considered the optimal ratio regarding dissolution performance during organic acid selection. As all prototypes were based on granules, the same batch of granules was used for all. Whenever possible, critical physical attributes (such as tablet porosity, for instance) were kept identical between prototypes. Table 2 summarises the similarities and differences between all prototypes.

### 2.3. Prototype Evaluation

#### 2.3.1. Biorelevant Dissolution Tests

Prototypes’ bio-performance was estimated via biorelevant dissolution tests in various media. The OrBiTo testing procedure for biorelevant dissolution was applied [29]. The different prototypes were analysed in biorelevant dissolution tests for 2 h in USP II at 37 °C with the paddle speed set at 50 rpm using peak vessels. A two-stage dissolution test was used, where the drug is first exposed to gastric medium for 30 min, then the transfer to the small intestine is simulated by adding a hybrid intestinal concentrate (buffer of FaSSIF-V1 and bile salt composition of FaSSIF-V2) at equal volume (final dissolution volume: 600 mL). As the target dose of 180 mg of API could not be achieved in a single unit for prototypes 1, 2 and 3, two capsules containing 90 mg of API were put in the dissolution bath to ensure comparability.

Biorelevant dissolution conditions were selected based on the in vitro study in dogs to evaluate the GiC with and without acid. Three gastric media were used in this study: a standard healthy gastric medium (FaSSGF), a low-buffered-capacity achlorhydric medium and a highly buffered achlorhydric medium (10 times buffer capacity). The compositions of the gastric media were based on previous studies [30,31]. The standard healthy gastric medium (FaSSGF) was composed of 300 mL pH 1.6 NaCl solution, with biorelevant surfactants. The low-buffered-capacity achlorhydric medium was composed of 300 mL of acetate/phosphate buffer 1 mM pH 7.0 with biorelevant surfactants, which was expected to be physiologically relevant for the target patient population. The highly buffered achlorhydric medium was composed of 300 mL of acetate/phosphate buffer 10 mM pH 7.0 with biorelevant surfactants and was selected for its high discriminative power. The starting intestinal medium was composed of 300 mL of FaSSIF double concentrate at pH 6.36 for achlorhydric conditions and pH 7.5 for the healthy condition.

Samples were taken at 0–5–10–20–29 min (gastric medium) and 35–40–50–60–90–120–150–180 min (intestinal medium). Samples were analysed using an in-house UPLC method. Results were expressed as a percentage of the dissolved dose of label claim. The dissolution tests were performed on six replicates, with the results displayed as mean and standard deviation.

#### 2.3.2. Bulk pH Measurement

The pH of the dissolution media was measured at the end of the gastric phase and at the end of the intestinal phase. In vivo, under physiological conditions, limited mixing within the mucus layer may allow a local pH gradient to persist even when the luminal bulk pH remains relatively unchanged. As the low-buffered-capacity medium provides little resistance to pH changes (similarly to the in vivo condition), and the media are continuously mixed during dissolution experiments (contrary to the in vivo condition), the locally generated pH change is rapidly propagated through the vessel. This in vitro artefact was leveraged, and the bulk pH was therefore used as an indirect indicator of the acidifying effect associated with drug dissolution and microenvironmental pH, rather than as a direct measurement of the pH at the particle surface.

This interpretation does not apply to the highly buffered medium. Its buffer capacity suppresses measurable bulk pH changes, while USP II agitation promotes rapid dissipation of local concentration and pH gradients. Thus, the bulk pH measured under this condition is unlikely to reflect the microenvironmental pH. This medium was, however, not intended to be physiologically representative and was included primarily as a discriminating condition for formulation prioritisation.

## 3. Results

The GiC prototypes with and without fumaric acid had been tested during a previous pharmacokinetic study in beagle dogs, demonstrating that the GiC containing fumaric acid allowed a 2.4-fold increase in the Cmax compared to the GiC formulation without fumaric acid [28]. Based on this proof of concept, different formulation strategies for incorporating a pH modifier in the formulation were evaluated in three biorelevant dissolution conditions to estimate their bioavailability.

### 3.1. Dissolution Behaviour in Highly Buffered Achlorhydric Media

The first screening was performed in highly buffered achlorhydric media (10 mM) to maximise the discrimination between prototypes. Dissolution results are shown in Figure 1.

The dissolution behaviour of the GiC (◊) and tablet (○) without acid was limited in this medium, while the GiC with acid (♦) presented a six-fold higher percentage dissolved at the beginning of the intestinal phase (35 min). The CiC (♦), bilayer tablet (▲), TiT (●), and mini-tablets in capsule (■) presented similar dissolution rates as the prototypes without acid (i.e., between 0.8 and 1.3 times the percentage dissolved after 35 min). For the bilayer tablet and mini-tablets in capsule, it was assumed that the fumaric acid and API were dispersed in the dissolution media before API solubilization due to the limited degree of contact between the API and acid. Moreover, as the media was highly buffered, acidification was prevented. For the CiC and TiT prototypes, it was observed that, during the dissolution, the inner tablet/capsule containing the acid did not disintegrate before the granule with API was completely dispersed in the media. These formulations could have been optimised to improve disintegration, which was not considered following positive results of other prototypes (see below). The monolayer tablet with acid (◍) allowed a higher initial dissolution of the API in the gastric compartment (i.e., 11.3% vs. 0.0% at 5 min). This was due to the faster disintegration of the tablet compared to the capsule. The rapid dissolution of the API in the gastric compartment was followed by a decrease in dissolved drug accompanied by turbidity, suggesting API reprecipitation. This behaviour is well documented for pH-sensitive poorly soluble drugs and is known to limit their bioavailability [32]. It resulted in a dissolved dose similar to prototypes without acid in the intestinal compartment. The TiC prototype with acid as powder (▬) led to a 1.98 times higher drug dissolution in the intestinal compartment after 35 min. However, the amount of fumaric acid had to be drastically increased (acid/API ratio 0.94) to achieve such a limited improvement. None of these prototypes demonstrated a similar or higher dissolution rate compared to the target GiC with acid. On the contrary, the over-encapsulated tablet (●) allowed achieving a percentage dissolved in the intestinal compartment 3.7 times higher than the GiC without acid. Interestingly, a parachute effect was also observed, leading to a percentage dissolved 5.1 times higher than the GiC without acid and 1.4 times higher than the GiC with acid after 120 min. The over-encapsulated tablet, therefore, showed the most favourable in vitro dissolution profile and was therefore selected for future in vivo evaluation.

The pH of the dissolution media was measured at the end of the gastric phase and at the end of the intestinal phase (Figure 2) to evaluate the ability of the prototypes to acidify the dissolution media. Only minor variations were observed, which was expected considering the buffer capacity of the dissolution media. Interestingly, this did not prevent the GiC with acid and the over-encapsulated tablet from achieving an enhanced dissolution rate. The disintegration mechanism of HPMC capsules was described by Dong et al. [33]. In an initial stage, the capsule shell exhibits hydration-induced swelling. This stage is followed by a rapid proliferation of micropores, allowing the infiltration of the dissolution medium inside the capsule, hence initiating the dissolution of the formulation components. Finally, the capsule shell disintegrates progressively, allowing the capsule content to be released and mixed within the media. Based on this mechanism, it was hypothesised that the water penetration during the second phase could induce the formation of an acidic microenvironment, hence promoting API dissolution inside the capsule shell. When the capsule finally disintegrates fully, the dissolved content is dispersed, and the pH is buffered. The API solubilization would nonetheless be maintained in the first stage of the intestinal phase.

### 3.2. Dissolution Behaviour in Low Buffered Capacity Achlorhydric Media

Following the tests performed in highly buffered achlorhydric media, a second screening was performed in low-buffered capacity achlorhydric media (1 mM). This medium has been reported to be more representative of the intragastric environment under hypochlorhydric conditions [30,31]. Dissolution results are shown in Figure 3.

In low-buffered conditions, the GiC without acid (◊) and tablet without acid (○) still presented reduced dissolution rates. Prototypes containing fumaric acid increased the percentage dissolved in the intestinal phase by 54 and 79 times compared to the prototypes without acid. When compared to the target, GiC with acid (♦) at 30 min, the bilayer tablet (▲) showed similar performance (i.e., 0.96 times the percentage dissolved). The monolayer tablet with acid (◍) and the over-encapsulated tablet with acid (●) performed similarly and slightly better than the GiC with acid (i.e., 0.95 times the percentage dissolved after 30 min and 1.4 times after 60 min). The TiC with acid as powder (▬) showed the highest dissolution rate in these dissolution conditions (i.e., 1.4 times at 30 min and 2.2 times at 60 min). Once again, this prototype contained a larger acid/API ratio compared to other prototypes with acid, which could explain the improved dissolution.

As explained in the Section 2, the bulk pH under low-buffered conditions was used as an indirect estimation of the microenvironment pH. When comparing the dissolution results with pH measurements taken at the end of the gastric and intestinal phases (Figure 4), a clear link was observed between the dissolution rate, the extent of pH reduction in the gastric phase and the acid/API ratio. Specifically, the TiC with acid as powder prototype, which contained a higher amount of acid, resulted in a more pronounced decrease in gastric pH. This result emphasised that, in a low-buffered-capacity achlorhydric medium, the dissolution rate was associated with the acidification of the dissolution media, which was not possible in highly buffered dissolution media. As a result, in low-buffered capacity media, increased dissolution rates are observed for all prototypes containing fumaric acid, regardless of the degree of contact between the API and the fumaric acid.

When comparing the results obtained between the low- and highly buffered achlorhydric media, the key difference was the reduced impact of the degree of contact between the API and acid, as especially observed with the bilayer tablet. This observation contradicted the initial hypothesis that a high degree of contact is needed to improve the dissolution throughout the creation of an acidic microenvironment. This result was explained by the acidification of the low- buffered media, which depended mainly on the acid/API ratio. As previously stated, the low-buffered media provides little resistance to pH changes, similar to the in vivo condition. The hydrodynamics, however, are not representative of the in vivo condition. It was therefore hypothesised that, in vivo, due to the limited agitation and low-buffered capacity in the stomach, the acidic microenvironment could indeed be formed regardless of the degree of contact between the API and organic acid. However, as there is still some mixing occurring in the stomach, the extent of microenvironment formation may be lower than in tested dissolution conditions. As a result, in vivo trials will be planned to evaluate prototypes with and without direct acid/API contact in a future study.

### 3.3. Dissolution Behaviour in Healthy Conditions

Based on dissolution tests in achlorhydric media, the GiC with and without acid, the tablet with and without acid and the over-encapsulated tablet prototypes were evaluated in biorelevant two-stage dissolution media mimicking healthy volunteers (Figure 5).

The tablet with (◍) and without acid (○) presented a higher dissolution in gastric media compared to the encapsulated prototypes, which was due to the relatively slow disintegration of the capsule shell. The over-encapsulated tablet (●) showed an even slower dissolution in the gastric phase, as the tablet could not fully disintegrate inside the capsule shell. Despite the observed differences in the gastric phase, all prototypes reached a similar asymptote in the intestinal media.

As expected, the pH measurements at the end of the gastric and intestinal phase presented only minor variation between prototypes (Figure 6). Selected prototypes therefore seemed suitable for patients without achlorhydria.

## 4. Discussion

Ten formulation prototypes were evaluated in biorelevant dissolution conditions to identify the best strategy for incorporating a pH modifier inside the formulation. As with all in vitro tests, assumptions were made about their bio-relevance. In particular, and as previously highlighted, hydrodynamics in dissolution vessels and in patients’ stomachs differ dramatically. Though in vivo tests would avoid making such hypotheses, in vitro testing allowed animal use to be reduced for such a large screening of formulation prototypes. By comparing the dissolution results in three dissolution conditions, it was possible to prioritise prototypes for future in vivo testing with reduced uncertainty.

To explain the improved dissolution rate observed in highly buffered media for the over-encapsulated tablet, it was assumed that the dissolution media first entered the capsule shell, hence creating an acidic microenvironment inside the capsule for the API to dissolve, which effect is maintained after capsule disintegration. This hypothesis was derived from research on the disintegration mechanism of HPMC capsules but was not formally evaluated and should be considered cautiously. Understanding the role of the capsule shell, and especially differences in behaviour in vivo compared to in vitro, would help strengthen the prototype selection.

Similarly, the differences observed between highly and low-buffered conditions were explained by the acidification of the dissolution media. While fumaric acid would disperse rapidly in the dissolution bath due to media agitation, it is expected to stay around the tablet or capsule in the stomach. As the stomach presents a low-buffered capacity, the creation of the acidic microenvironment is expected to take place in vivo even without the capsule to maintain the API and fumaric acid together for dissolution enhancement. In this study, the bulk pH of the low-buffer dissolution media was hypothesised to be representative of the microenvironmental pH occurring in the stomach. Being able to monitor microenvironmental pH during in vivo studies and designing representative biorelevant tests specifically to support formulation strategies based on pH modification would help to assess prototypes.

Finally, formulation selection should not only consider bio-performance, but also drug product stability, manufacturability, and patient-centricity. In this study, the stability risk was mitigated by the selection of the organic acid. Regarding drug product stability, the different prototypes were also evaluated under stress conditions (i.e., open dish, 40 °C/75%RH), confirming improved drug product stability when the API and fumaric acid are not in direct contact (degradation only observed at the interface or not if both entities were well separated). However, as instability induced by organic acid is based on moisture adsorption, protective packaging may also be considered to improve product stability without impairing manufacturability and bio-performance. In terms of manufacturability, all prototypes could be produced successfully at laboratory scale. It should nonetheless be noted that a successful prototype would then have to be industrialised. While the GiC and monolayer tablets would be easily scalable, the bilayer tablet, TiT and minitablets in capsules require specific tooling and potentially reduced manufacturing speed. The over-encapsulated tablet and TiC with acid as powder require an additional process step with specific equipment as well. In terms of patient-centricity, the target dose could not fit into a single size 1 capsule for prototypes in capsule (i.e., GiC, CiC, over-encapsulated tablet, minitablets in capsule and TiC with acid as powder). This may negatively impact patient adherence by increasing pill burden. Prototypes developed in this study were, however, not optimised in terms of shape and dimensions. While the GiC, CiC and minitablets in capsule prototypes required either two size 1 capsules or one size 00 capsule to fit the target dose, the over-encapsulated tablet and the TiC with acid as powder could be optimised to fit into a size 1 capsule.

## 5. Conclusions

In this study, 10 formulation strategies were evaluated in biorelevant dissolution media to select the best approach for incorporating a pH modifier into an oral solid dosage form. The goal was to improve the dissolution profile of the drug in biorelevant conditions with acceptable amounts of pH modifier to ensure manufacturability and reduced capsule/tablet size for improved patient experience.

In vitro data showed that the over-encapsulated tablet prototype led to a percentage dissolved in the intestinal media up to 5.1 times higher than the GiC without acid and 1.4 times higher than the GiC with acid in highly buffered achlorhydric conditions. This prototype was considered the most promising formulation toward elevated gastric pH. However, in low-buffered achlorhydric conditions, the monolayer tablet with acid exhibited a dissolution rate similar to the over-encapsulated tablet (i.e., up to 54 times compared to the GiC without acid and 1.4 times compared to the GiC with acid). It was also noticed that the bilayer tablet, characterised by a limited contact between the API and the organic acid, also led to a percentage dissolved up to 73 times higher than the GiC without acid. This result suggested that the acidic microenvironment may not require such a high degree of contact between the API and the pH modifier. The outcomes of this study, and consequently the bio-relevance of tested media, will be further evaluated in vivo. For these tests, the over-encapsulated tablet with acid and the monolayer tablet with acid will be evaluated together with the GiC and monolayer tablet without acid in pre-treated healthy volunteers to determine whether the formulations improve systemic exposure or relative bioavailability.

## Figures and Tables

**Figure 1:**
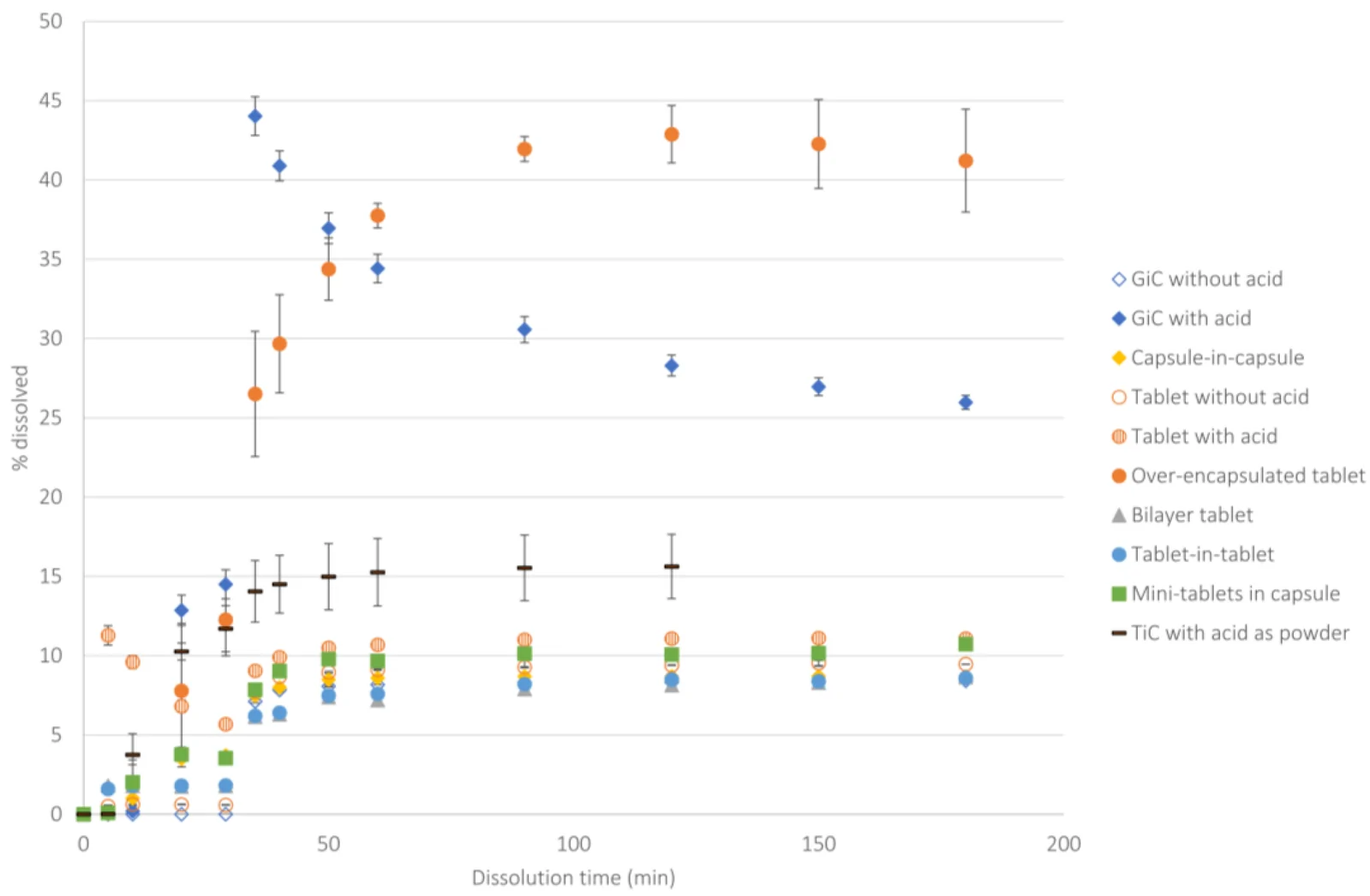
Two-stage dissolution results in highly buffered achlorhydric media.

**Figure 2:**
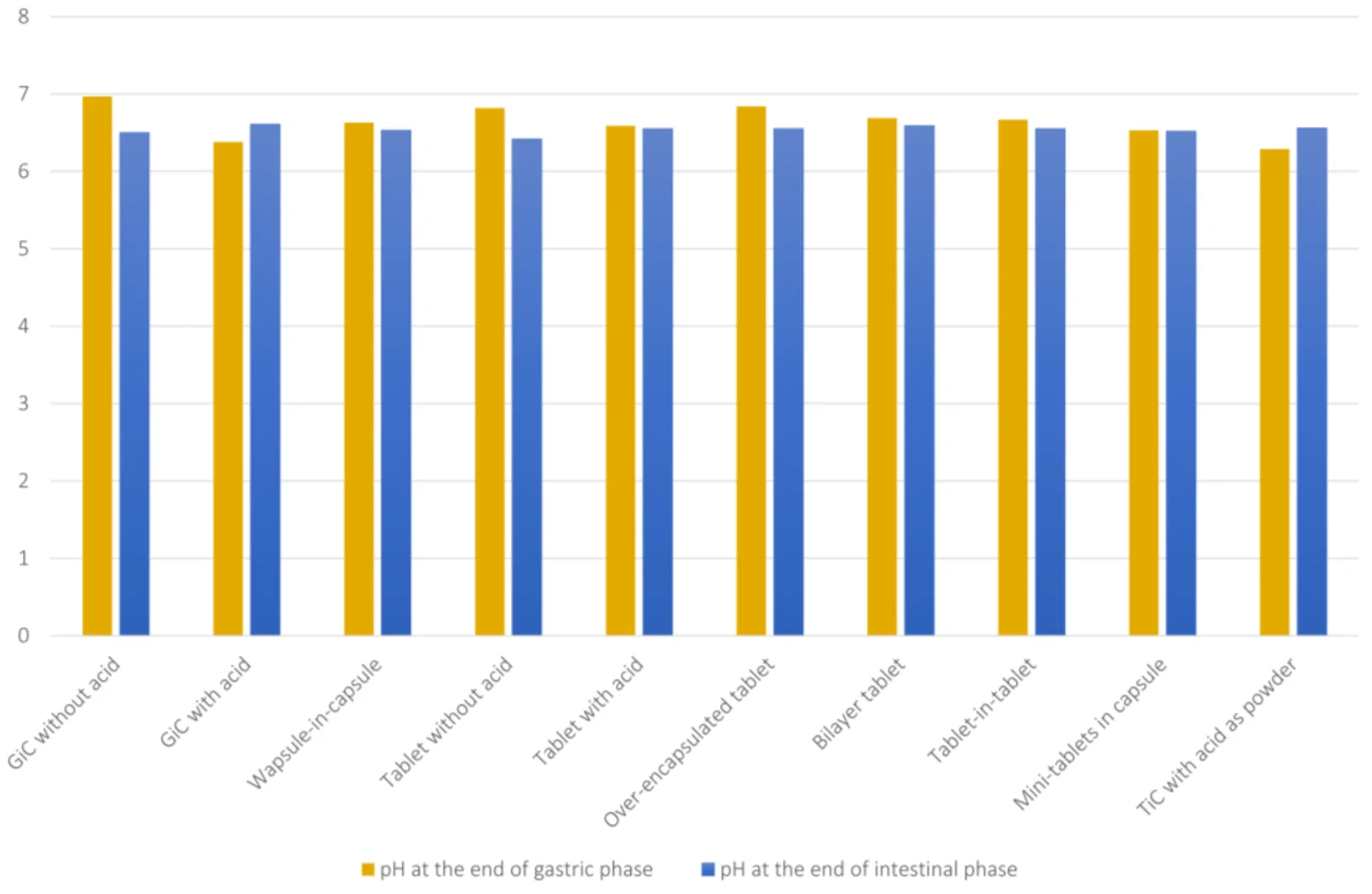
pH measurements at the end of the gastric and intestinal phase in highly buffered achlorhydria media.

**Figure 3:**
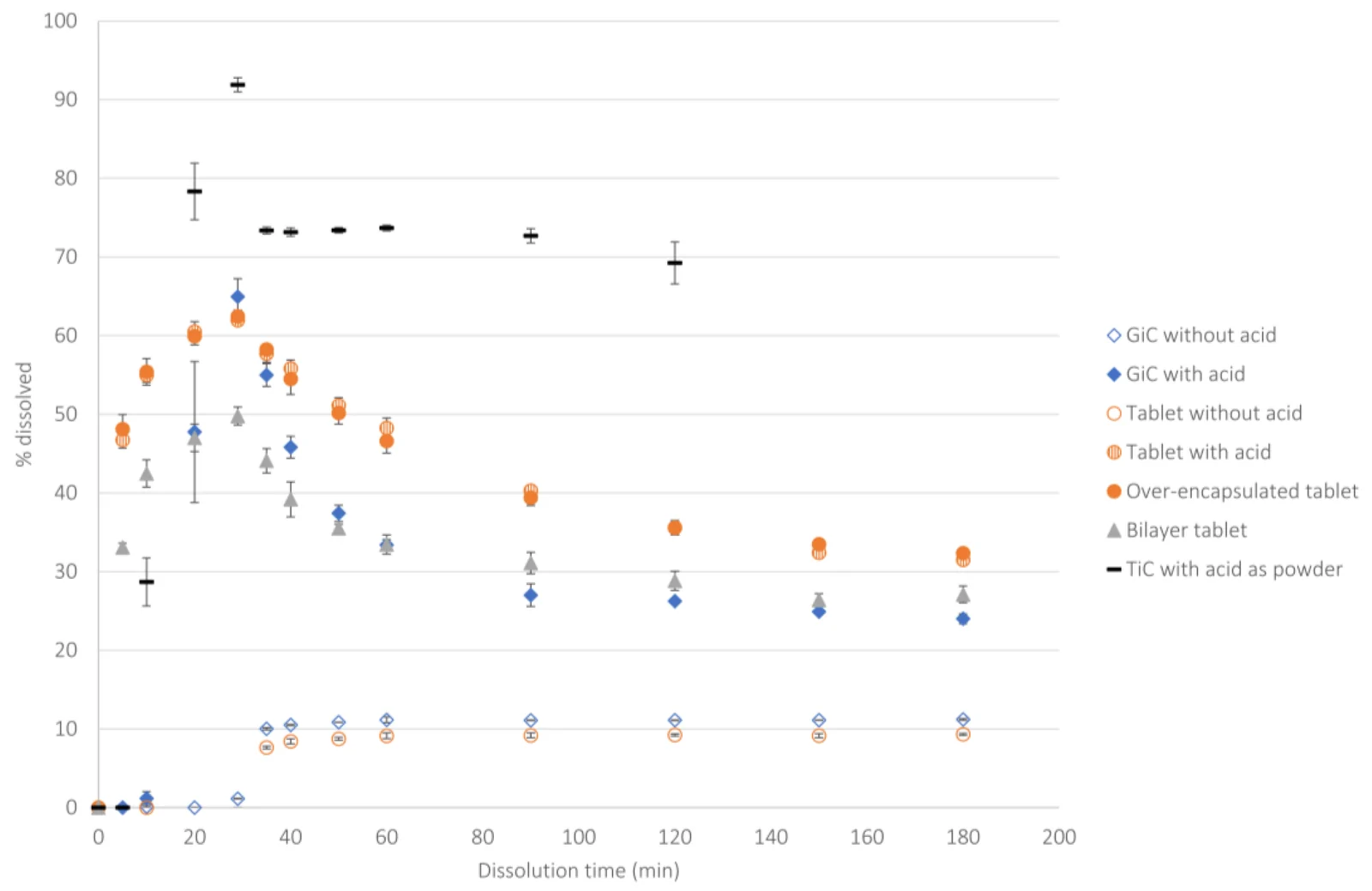
Two-stage dissolution results in low-buffered capacity achlorhydric media.

**Figure 4:**
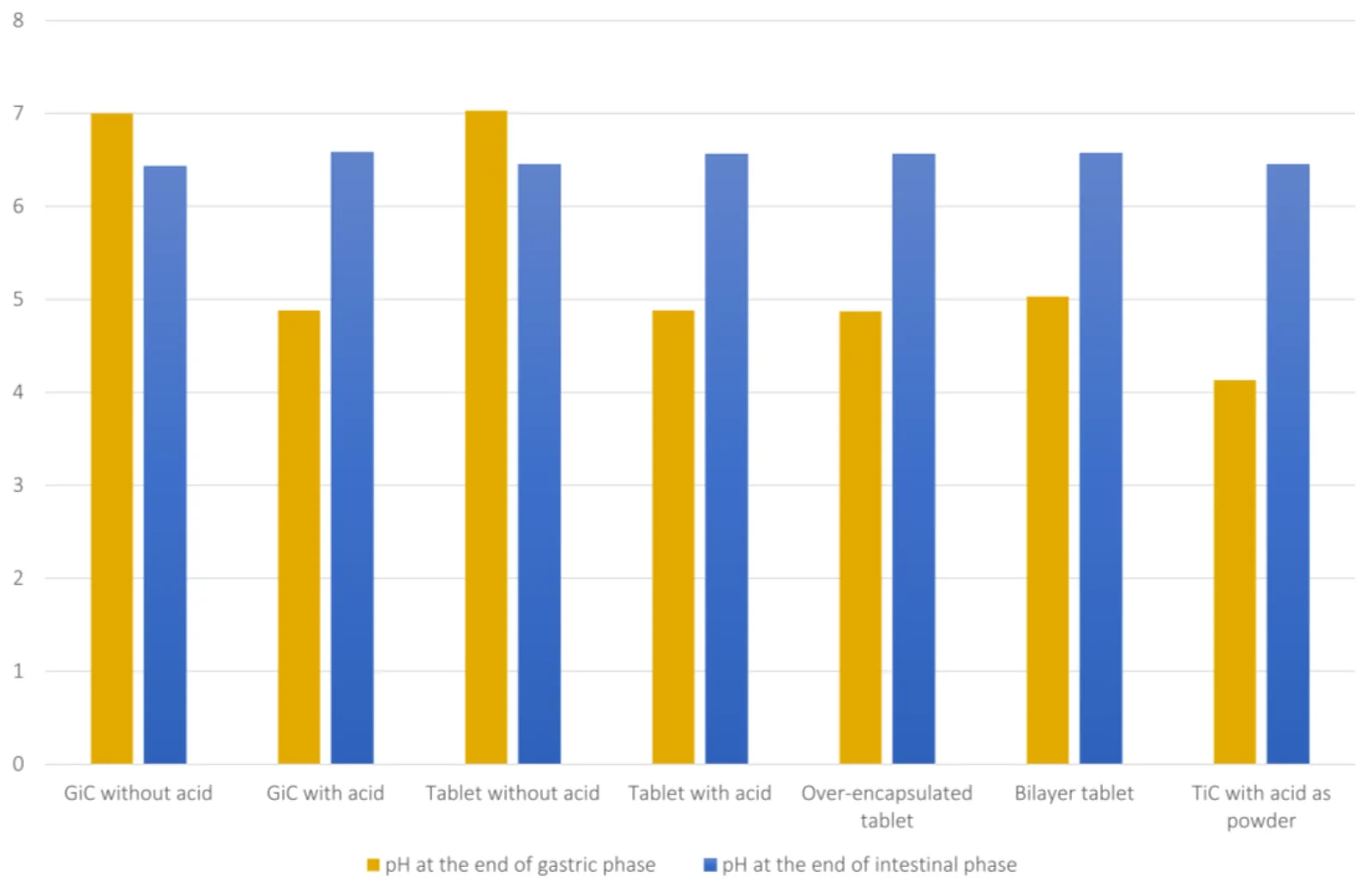
pH measurements at the end of the gastric and intestinal phase in low-buffered capacity achlorhydria media.

**Figure 5:**
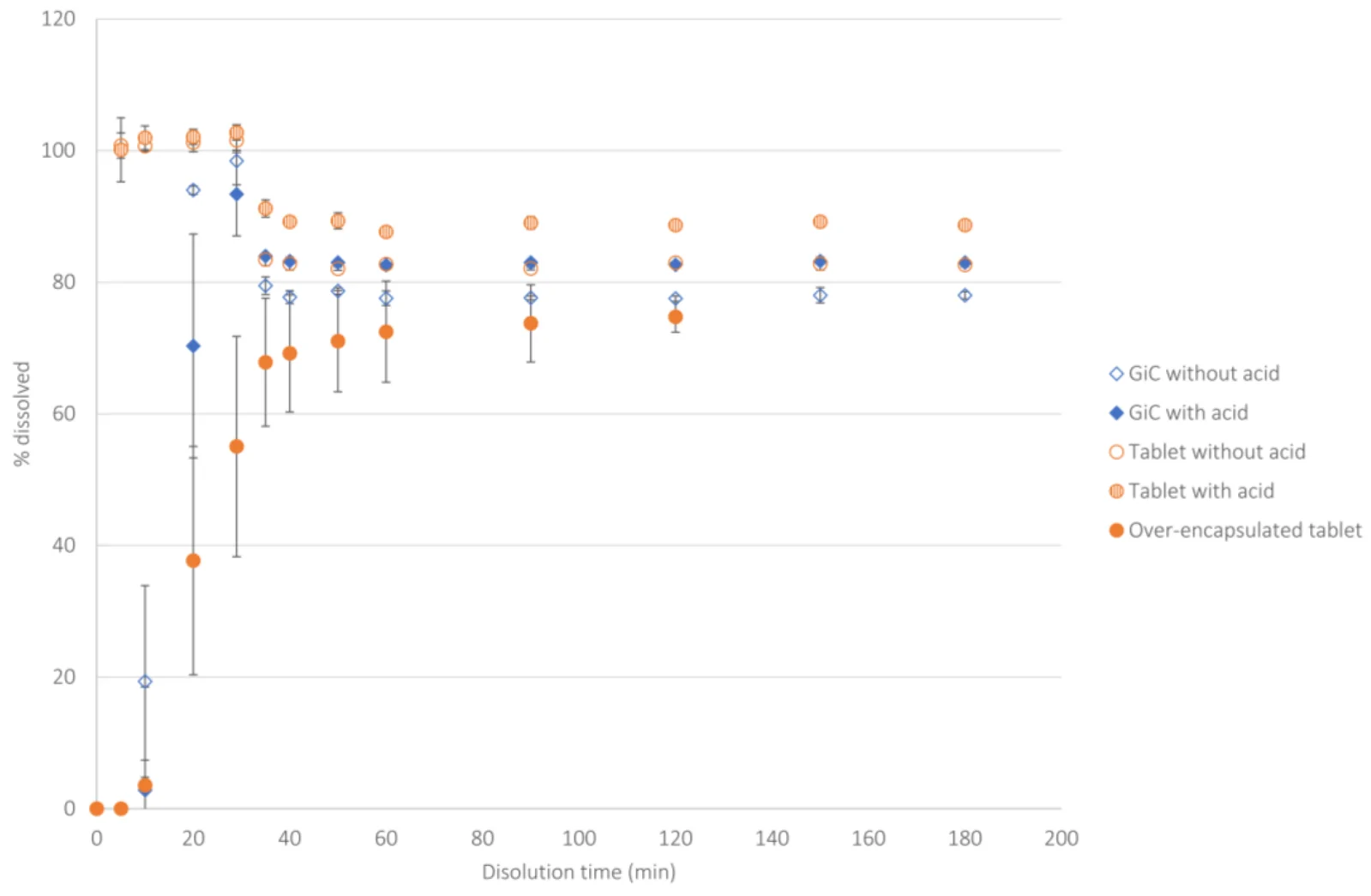
Two-stage dissolution results in healthy conditions.

**Figure 6:**
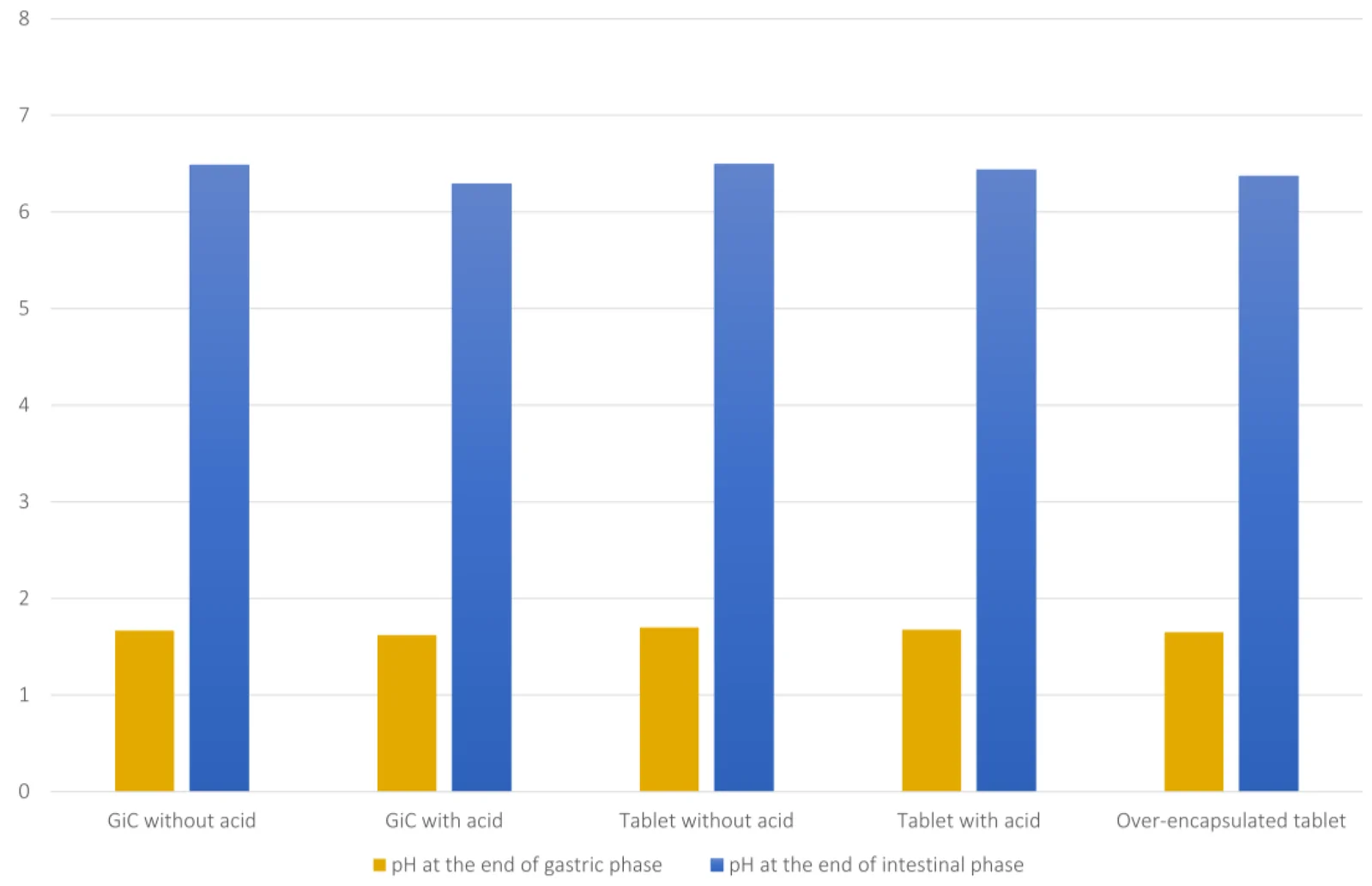
pH measurements at the end of the gastric and intestinal phase in healthy conditions.

**Table 1:** Description of developed formulations.

#	Prototype Name	Description	Picture
1	GiC without acid	Granules-in-Capsule (GiC) without acid	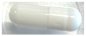
2	GiC with acid	GiC with acid in the extra-granular phase	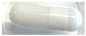
3	CiC	Capsule-in-Capsule (CiC) with acid in the inner capsule and API in the outer capsule	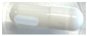
4	Tablet without acid	Monolayer tablet without acid	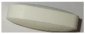
5	Tablet with acid	Monolayer tablet with acid in the extra-granular phase	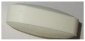
6	Over-encapsulated tablet	Over-encapsulated tablet with acid in the extra-granular phase	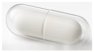
7	Bilayer tablet	Bilayer tablet with an active layer and a layer with acid	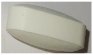
8	TiT	Tablet-in-Tablet (TiT) with an acid core tablet and an active shell	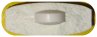
9	Mini-tablets in capsule	Mini-tablets in capsule with acid and active mini-tablets	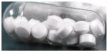
10	TiC with acid as powder	Tablet (without acid) and acid as powder in capsule	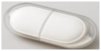

**Table 2:** Comparison of developed formulations.

#	Prototype Name	API Amount (mg)	Acid/API Ratio	Unit Weight (mg)	Dimensions
1	GiC without acid	90	0.00	226.1	Size 1
2	GiC with acid	90	0.22	246.1	Size 1
3	CiC	90	0.22	272.8	Size 00 & 4
4	Tablet without acid	180	0.00	533.0	16 × 7.37 mm
5	Tablet with acid	180	0.22	533.0	16 × 7.37 mm
6	Over-encapsulated tablet	180	0.22	533.0	16 × 7.37 mm/Size 0
7	Bilayer tablet	180	0.22	665.0	14.48 × 8.13 mm
8	TiT	180	0.22	577.9	5.5 in 16 × 7.37 mm
9	Mini-tablets in capsule	180	0.22	587.9	2.5 mm/Size 00
10	TiC with acid as powder	180	0.94	702.0	16 × 7.37 mm/Size 00

## Data Availability

The original contributions presented in this study are included in the article. Further inquiries can be directed to the corresponding author(s).

## Abbreviations

The following abbreviations are used in this manuscript:

API	Active Pharmaceutical Ingredient
BCS	Biopharmaceutical Classification System
CiC	Capsule-in-Capsule
GiC	Granules-in-Capsule
HPC	Hydroxy-Propyl Cellulose
MCC	Microcrystalline Cellulose
TiC	Tablet-in-Capsule
TiT	Tablet-in-Tablet
